# Optogenetic activation of axon guidance receptors controls direction of neurite outgrowth

**DOI:** 10.1038/srep23976

**Published:** 2016-04-07

**Authors:** M. Endo, M. Hattori, H. Toriyabe, H. Ohno, H. Kamiguchi, Y. Iino, T. Ozawa

**Affiliations:** 1 Department of Chemistry, Graduate School of Science, The University of Tokyo, 7-3-1 Hongo, Bunkyo-ku, Tokyo 113-0033, Japan; 2 Department of Biological Sciences, Graduate School of Science, The University of Tokyo, 7-3-1 Hongo, Bunkyo-ku, Tokyo 113-0033, Japan.; 3 Laboratory for Neuronal Growth Mechanisms, Brain Science Institute, The Institute of Physical and Chemical Research (RIKEN), 2-1 Hirosawa, Wako, Saitama 351-0198, Japan

## Abstract

Growth cones of extending axons navigate to correct targets by sensing a guidance cue gradient via membrane protein receptors. Although most signaling mechanisms have been clarified using an *in vitro* approach, it is still difficult to investigate the growth cone behavior in complicated extracellular environment of living animals due to the lack of tools. We develop a system for the light-dependent activation of a guidance receptor, Deleted in Colorectal Cancer (DCC), using *Arabidopsis thaliana* Cryptochrome 2, which oligomerizes upon blue-light absorption. Blue-light illumination transiently activates DCC via its oligomerization, which initiates downstream signaling in the illuminated subcellular region. The extending axons are attracted by illumination in cultured chick dorsal root ganglion neurons. Moreover, light-mediated navigation of the growth cones is achieved in living *Caenorhabditis elegans*. The photo-manipulation system is applicable to investigate the relationship between the growth cone behavior and its surrounding environment in living tissue.

Precise assembly of functional neural circuits requires strictly regulated sequential wiring of individual axons during nervous system development. Disruption of the axon pathfinding by neurodegenerative disorders engenders severe impairments in both motor and cognitive functions^1^,^2^. For the regeneration of a damaged nervous system, it is crucially important to understand the inherent regulatory mechanisms of the neuronal wiring. In developing brains, extending axons are navigated to their correct targets by multiple extracellular guidance cues secreted or exhibited by the surrounding cells^3^. An axon recognizes the cues with their receptors on the growth cone, dynamic actin-rich structure at the nerve ending^4^. The cue-induced receptor activation triggers downstream attractive or repulsive signaling cascades, resulting in cytoskeletal remodeling of growth cone to turn the extending axons in the direction of their correct targets. To date, the dynamic behaviors of the growth cone have been investigated mainly in cultured primary neurons, using established *in vitro* experimental techniques such as asymmetric chemical gradients around the growth cone^5^,^6^,^7^,^8^. However, the developing axons in an embryo are surrounded by a highly complicated environment with heterogeneous extracellular matrix and cellular barriers, which strongly affect the axon pathfinding. To assess the dynamic influence of the surrounding complex milieu on the developing axons during their pathfinding *in vivo*, a novel method is necessary for the direct manipulation of the axon guidance signaling in living animals.

Optical modulation of protein activity has gained attention as a promising approach to dissect various biological phenomena in intact organisms, represented by functional analysis of neural circuits with light-gated ion channels^9^,^10^. Recently, some receptors and kinase proteins have also been converted into photo-activatable forms using plant or bacterial photoreceptor proteins, which change their conformation upon light absorption at a specific wavelength^11^,^12^,^13^,^14^. Unlike activation with natural or synthetic ligand injection, which has low spatiotemporal precision due to their diffusion, rapid and local light delivery enables precise photo-activation of the target proteins with millisecond and subcellular resolution. Therefore, photoreceptor proteins have great potential for unveiling the intrinsic dynamics of biological events in living animals. Cryptochrome 2 (CRY2), a photoreceptor protein derived from *Arabidopsis thaliana*, forms a cluster upon blue light (440–488 nm) illumination^15^. Photo-clustering of CRY2 is applicable for the oligomerization and activation of proteins, thereby providing us a new strategy to generate photo-activatable proteins^16^,^17^,^18^,^19^.

Here, we show a photo-activation system for DCC, an axon guidance receptor protein embedded in the plasma membrane. Cytoplasmic domain of DCC oligomerizes upon its extracellular ligand binding, which is crucial for the activation of intracellular signaling to induce cytoskeletal remodeling^20^. To control the oligomerization by external light, CRY2 is connected genetically with DCC, named photo-activatable DCC (PA-DCC). PA-DCC enables us to induce axon attraction by blue light illumination in chick DRG neurons. Finally, we demonstrate its applicability for the *in vivo* light-induction of growth cone turning in the nematode *Caenorhabditis elegans*.

## Results

### Light-dependent activation of PA-DCC molecules

**Figure 1:**
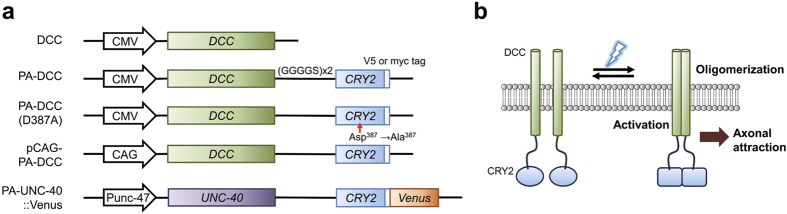
Schematics for the structures and photo-activation model of PA-DCC. (**a**) Domain structures of PA-DCC molecules. All text in italics refers to the genes of their corresponding proteins. CMV, CAG and Punc-47 represent promoters for gene expression. DCC or UNC-40 is connected with CRY2 via GS linker (GGRGGGGSGGGGS). Venus is a yellow fluorescent protein whose sequence is optimized for *C. elegans* expression. White squares show epitope V5 or myc tags. (**b**) Schematic diagram for the strategy of DCC activation with blue light illumination.

Reportedly, the attachment of GFP to the N-terminus of CRY2 reduces weak or transient self-association with endogenous CRY2 in dark without disturbing light-induced cluster formation^15^,^21^. To reduce the basal oligomerization level of the engineered DCC protein in a dark state, the cytoplasmic tail of DCC was connected with the amino-terminal end of CRY2 via a flexible GS linker (GGRGGGGSGGGGS), resulting in a protein named PA-DCC (Fig. 1a). Because of the light-dependent CRY2 oligomerization, we inferred that fused DCCs might undergo oligomerization and consequent activation, leading to axonal attraction (Fig. 1b).

**Figure 2:**
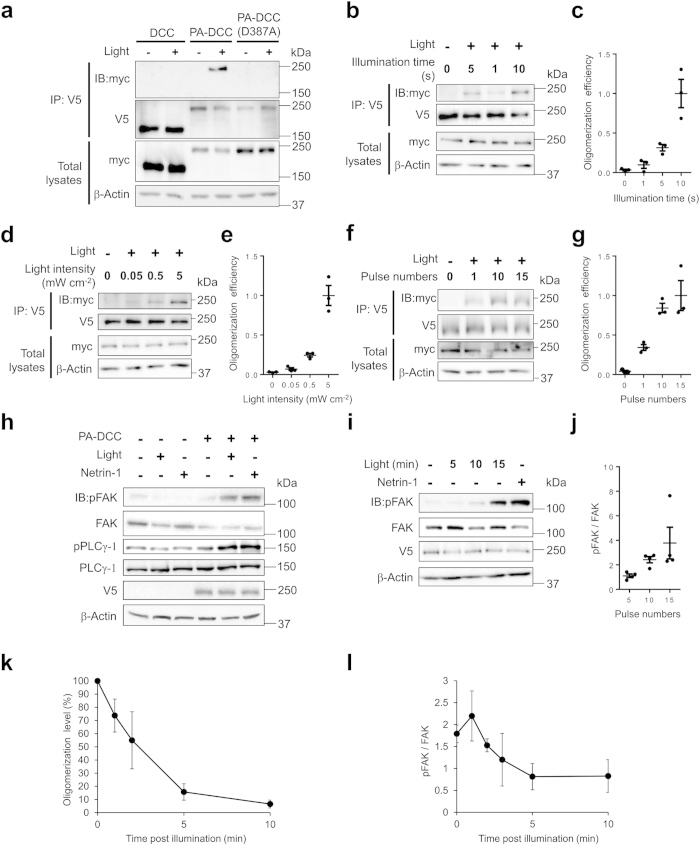
Activation of PA-DCC with blue light. (**a**) Confirmation of DCC oligomerization. Cells co-expressing myc-tagged and V5-tagged molecules were lysed after illumination (10 s min^−1^ pulses for 15 min). V5-tagged molecules were immunoprecipitated with the anti-V5 antibody. (**b,c**) Illumination time dependency of PA-DCC oligomerization. Cells were lysed after illumination (1, 5 or 10 s min^−1^ pulses for 15 min). A representative data was shown in (**b**). (**c**) The efficiencies were plotted as scores relative to the maximal value. Error bars, SEM (*n* = 3). (**d,e**) Light intensity dependency of the oligomerization. Cells were lysed after illumination (10 s min^−1^ pulses for 15 min). Error bars, SEM (*n* = 3). (**f,g**) Pulse number dependency of PA-DCC oligomerization. Cells were lysed after illumination (10 s min^−1^ pulses for 15 min). Error bars, SEM (*n* = 3). (**h**) Analysis of the phosphorylation of FAK and PLCγ-1. Cells were stimulated with light or with netrin-1 (200 ng mL^−1^) for 15 min. (**i,j**) Pulse number dependency of the FAK phosphorylation. Cells were lysed after illumination (10 s min^−1^ pulses for 1, 10 or 15 min). Phosphorylation level was indicated as scores relative to the basal level. Error bars, SEM (*n* = 4). (**k**) Dissociation profile of PA-DCC oligomer. The oligomerization level was indicated relative to the level just after the illumination. Error bars, SD (*n* = 3). (**l**) Dephosphorylation of FAK after light illumination. The phosphorylation level was indicated relative to the basal level. Error bars, SD (*n* = 3).

To investigate whether PA-DCC is oligomerized upon light stimulation, myc or V5 epitope tag was attached to the PA-DCC for co-immunoprecipitation (co-IP). DCC connected with photo-insensitive CRY2 mutant (D387A)^22^, represented as PA-DCC (D387A), and DCC without CRY2 (DCC) were also prepared as a non-light reactive control (Fig. 1a). Each pair of the tagged molecules was coexpressed in HEK293T cells, which do not express endogenous DCC^23^. Plasma membrane localization of the molecules was confirmed using confocal microscopy (Fig. S1). Cells coexpressing the tagged molecules were exposed to blue light (5 s min^−1^) for 15 min before co-IP assay. The protein samples of IP by anti-V5 antibody were analyzed to examine whether the myc-tagged molecules were co-immunoprecipitated (Fig. 2a). Myc-tagged molecules were pulled down with V5-tagged molecules upon the blue light illumination. In the case of DCC and PA-DCC (D387A), the myc-tagged molecules were not detected. The light-induced oligomerization efficiency of PA-DCC, defined as the myc band intensities normalized by the V5 band intensities in the immunoprecipitated samples, increased along with the illumination time (Fig. 2b,c), with light intensity (Fig. 2e,f), and with the pulse number (Fig. 2g,h). Light-induced PA-DCC oligomerization was not observed under the epi-fluorescence microscope (data not shown), which suggests that the size of the PA-DCC oligomer is smaller than the optical resolution. Collectively, these results indicate that photo-conversion of CRY2 triggered PA-DCC oligomerization.

Netrin-1, a major attractive guidance molecule, binds to an extracellular domain of DCC, resulting in DCC oligomerization^24^,^25^. A cytoplasmic P3 domain of DCC mediates its oligomerization and also interacts with signaling proteins such as Focal Adhesion Kinase (FAK)^26^ and phosphatidylinositol transfer protein α^27^. Upon netrin-1 binding, these interactions respectively trigger the phosphorylation of FAK and PLCγ-1, initiating the downstream signaling cascade for axon turning. To investigate whether light-dependent oligomerization of PA-DCC trigger the signaling, phosphorylation levels of FAK and PLCγ-1 were examined. Results confirmed that FAK and PLCγ-1 in PA-DCC expressing cells were phosphorylated with blue light illumination, although light illumination on the cells without PA-DCC expression did not induce their phosphorylation (Fig. 2h). Additionally, the phosphorylation levels of FAK elevated with increasing number of light pulses (Fig. 2i,j), indicating that the extent of the activation is tunable by the light dose. These results demonstrate that light-induced CRY2 oligomerization can trigger the DCC activation.

### Reversibility of PA-DCC activation

To confirm the reversibility of light-dependent activation of PA-DCC, the dissociation profile after light illumination of PA-DCC was investigated. Light-exposed cells expressing PA-DCC were incubated in the dark before assays. The results of co-IP showed that oligomerized PA-DCCs disappeared along with the elapsed time after illumination (Fig. 2k, Fig. S2a). Most PA-DCCs returned to monomer within 10 min. Accordingly, the phosphorylated FAK in the cells decreased gradually over time (Fig. 2l, Fig. S2b). The time course of PA-DCC deactivation corresponded to that of the dissociation of the PA-DCC molecules after the illumination. These results indicate that blue light illumination activates PA-DCC transiently, thereby ensuring the capability of the tool to guide axons to the desired direction with repetitive illumination on growth cones.

### Axonal attraction by light-induced PA-DCC activation

**Figure 3:**
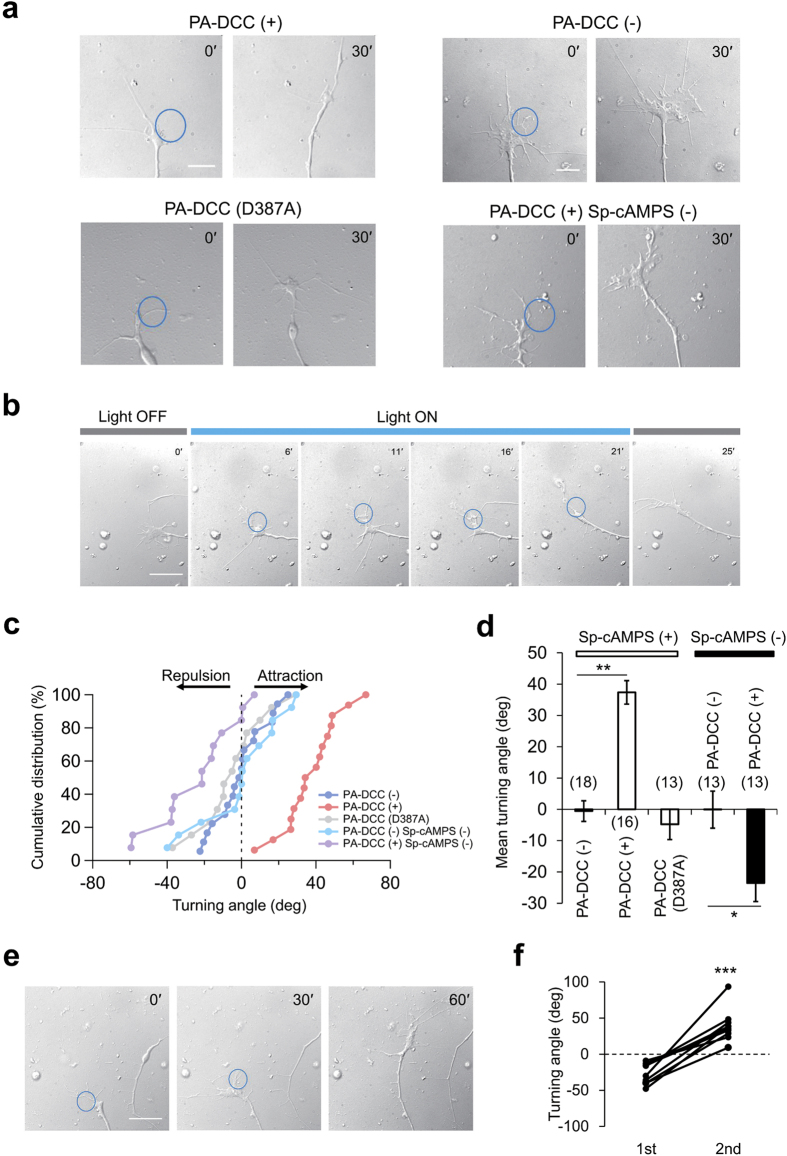
Light-induced growth cone turning of chick DRG neurons expressing PA-DCC. (**a**) DIC images of illuminated growth cones. Blue circles represent illuminated regions (5 s every 5 min pulses). The images were taken at initial time (0΄) and 30 min after (30΄). Scale bar, 5 μm. (**b**) Time lapse images of light-induced growth cone attraction with PA-DCC. Digits show elapsed minutes. The illuminations were performed at *t* = 6, 11, 16, 21 min. Scale bar, 10 μm. (**c**) Cumulative distribution of final growth cone turning angles in the light-induced growth cone attraction assay. Turning angles were calculated based on the original definition explained in Methods section. (**d**) Turning angles of growth cones with illumination. Numbers in parentheses indicated the total number of growth cones tested. Error bars, SEM. Statistical significance was determined by one-way ANOVA followed by Dunnett’s test for multiple comparison, **P < 0.01; or by *t-*test, *P < 0.05. (**e**) Repositioned illuminations caused repeated light-induced growth cone attraction to different directions. Scale bar, 10 μm. (**f**) Turning angles of each growth cone before and after repositioned illuminations. Ten growth cones were examined. ***P < 0.005, paired *t*-test.

**Figure 4:**
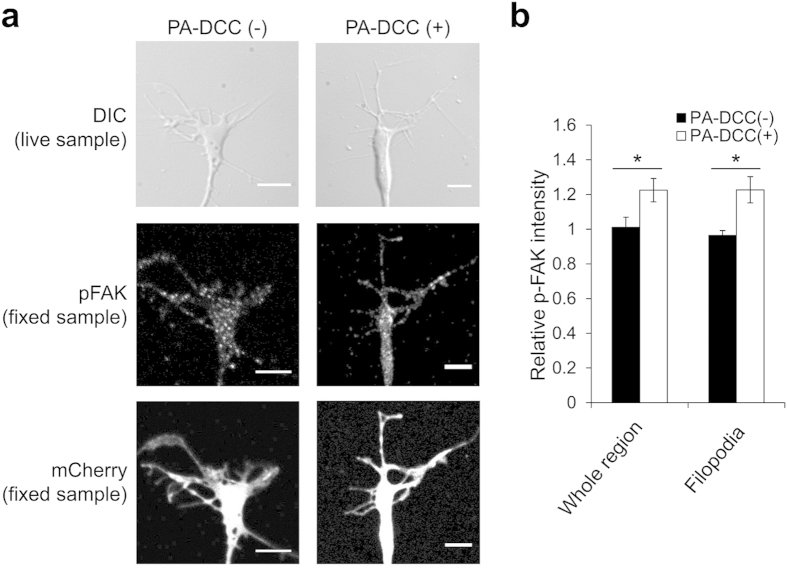
Local phosphorylation of FAK. (**a**) Transmitted, fluorescence and immunostained images of growth cones. A part of chick DRG growth cones with or without PA-DCC was illuminated with blue light (5 s every 5 min pulses for 15 min). Growth cones were fixed just after the third illumination and immunostained with the specific antibody. The right sides of the growth cones were illuminated. Scale bar, 5 μm. (**b**) Analysis of FAK phosphorylation level in the illuminated side. Mean phosphorylation intensity in the illuminated side was indicated as scores relative to the intensity in the non-illuminated side. pFAK intensity was normalized by mCherry fluorescence. Error bars, SEM (*n* = 5). *P < 0.05, unpaired *t*-test.

To explore the possibility of the axonal attraction with PA-DCC, growth cone turning assays were performed in DRG neurons from chick embryos (E9). cDNA encoding PA-DCC under control of the CAG promoter (Fig. 1a) was transfected to DRG neurons. It was confirmed that PA-DCC localized on the cellular membrane at the area of growth cone (Fig. S3). The transfected DRG neurons were plated on the laminin-1 coated dishes. To suppress the influence of laminin-1-dependent decreases in the cytoplasmic cAMP/cGMP level in the growth cones, the neurons were treated with Sp-cAMPS, an activator of cAMP-dependent kinase, before each assay^28^,^29^,^30^. A part of transfected growth cones was illuminated with blue light in a pulse-like manner (5 s every 5 min) during the observation. Light-induced axonal attraction was clearly observed in neurons expressing PA-DCC (Fig. 3a). In contrast, the blue light illumination on the growth cones without PA-DCC expression or with light-insensitive PA-DCC (D387A) mutant did not affect the axonal growth direction. Time lapse imaging of the light-induced axonal attraction revealed the clear growth cone movements in response to the pulsatile illuminations (Fig. 3b and Movie S1). Figure 3c presents a cumulative distribution of final growth cone turning angles. In most of the assays (14 out of 16), growth cones expressing PA-DCC turned at an angle of greater than 25°. The mean turning angle of growth cones with PA-DCC expression was 37.4 ± 3.8°, which was significantly different from that of growth cones without PA-DCC expression (−0.6 ± 3.3°) (Fig. 3d). After the light-induced attraction, the illumination area was repositioned from the left side to the right side. Also, the pulse-like illuminations continued further (Fig. 3e). The turning angles shown are depicted in Fig. 3f and Fig. S4, where positive values represented growth cones turning to the right side. Statistical analysis revealed that all examined growth cones, which were attracted by the first illumination at the angles of −26.2 ± 4.4°, turned to the repositioned illuminated side at the angles of 39.2 ± 7.0° upon subsequent illumination. The results demonstrate the capability of redirecting the growth cone with repeated light-induced turning. To confirm that the illumination on a part of the growth cone triggered attractive signaling locally, phosphorylation of FAK within illuminated growth cones were examined by immunocytochemistry (Fig. 4a). Compared to the growth cone without PA-DCC, FAK phosphorylation signals increased in the illuminated side, including in filopodia (Fig. 4b). In the absence of Sp-cAMPS treatment, light-induced axon repulsion was observed (Fig. 3a,c,d). Considering that the reduction of the cAMP/cGMP level converts axonal attraction to axon repulsion in netrin-1/DCC signaling^29^, the obtained results collectively suggest that CRY2-mediated oligomerization of PA-DCC upon light illumination triggered the downstream signaling pathway of DCC. Therefore, PA-DCC can control the neurite outgrowth direction with light illumination in chick DRG neurons.

### *In vivo* photo-manipulation of the growth cone behavior in *C. elegans*

Finally, we investigated whether it is possible to control the direction of axon extension with light in living organisms. The nervous system in nematode worm *C. elegans* has several features that are beneficial for the experiment: its cell lineage and its neural connections have been anatomically identified; also, it shares common guidance mechanisms with other species, including mammals. Of several neuron types in *C. elegans*, GABAergic VD motor neurons are guided by UNC-6/netrin in the post-embryonic stage. The behavior of their growth cones in the neural network establishment stage has been analyzed using time-lapse imaging^31^,^32^. For these reasons, VD neurons in *C. elegans* were selected as a target for the *in vivo* photo-manipulation of the growth cone behavior.

The amino acid sequence of UNC-40, a *C. elegans* homolog of DCC, exhibits low homology to vertebrate DCC in the intracellular domain^33^. To improve the efficiency of light-induced signal transduction in *C. elegans* cells, DCC in PA-DCC was replaced with UNC-40, resulting in a protein named PA-UNC-40. In addition, the carboxy terminus of PA-UNC-40 was fused with a fluorescent protein of Venus for examining its expression (Fig. 1a). The gene of PA-UNC-40::Venus was placed under the *unc-47* promoter for the expression in GABAergic neurons including VD neurons^34^.

In *C. elegans*, UNC-6 is secreted from ventral cells, which is necessary for the circumferential axon guidance^35^. VD axons express both UNC-40 and UNC-5, which cooperates with UNC-40 to mediate repulsive response to UNC-6, thereby extending dorsally at the L2 stage^31^,^36^. To eliminate interference with the endogenous guidance system in VD axons, a mutant worm lacking both *unc-5* and *unc-6* (*unc-5(e53); unc-6(ev400)*) was generated. Consistent with the previous report^36^, almost all VD axons failed to reach the dorsal nerve cord in the mutant (Fig. S5a,b). Subsequently, cDNAs of PA-UNC-40::Venus and mCherry were introduced into the mutant and a transgenic line was obtained. The PA-UNC-40::Venus molecules distributed uniformly on the VD growth cone membrane (Fig. S6a,b), which was similar to the localization pattern of UNC-40 in the *unc-6* null mutant background^37^. The PA-UNC-40 expression in the mutant did not rescue the axon guidance defects in VD axons (Fig. S5a,b).

**Figure 5:**
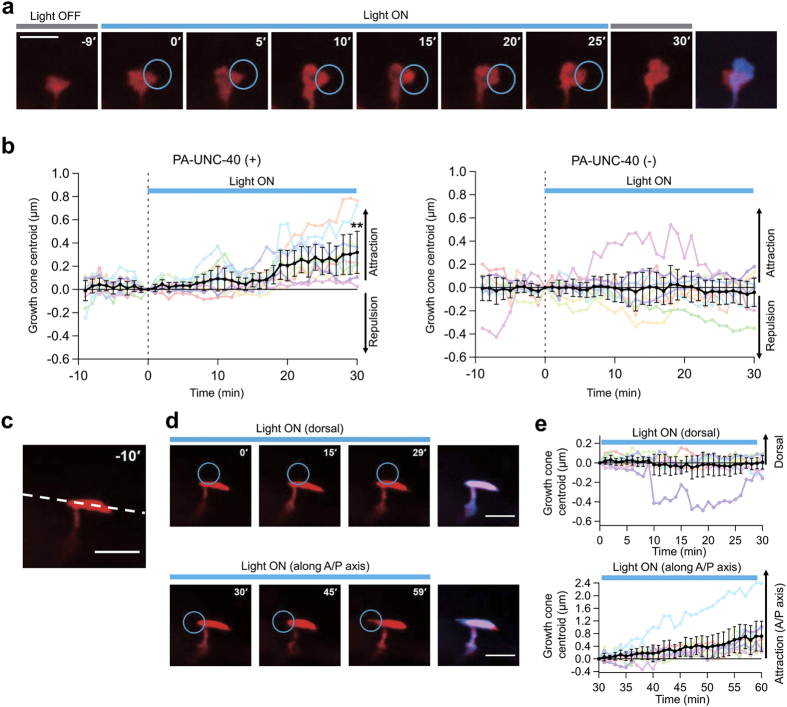
Light-induced growth cone attraction in *C. elegans* expressing PA-UNC-40. (**a**) Time lapse images showing light-induced VD growth cone attraction. Blue circles represents illuminated regions with laser light (2 s min^−1^ pulses, 488 nm). The merged image (Red; 0 min, Blue; 30 min) was attached. Scale bar, 3 μm. (**b**) Temporal centroid plots of periodically illuminated growth cones. Worm strains used were *unc-5(e53); unc-6(ev400)* with or without PA-UNC-40::Venus expression. Each data is represented by a colored line. Averaged data are shown as black. Error bars, 98% CI of the mean (*n* = 10). **P < 0.01 versus PA-UNC-40 (−) at 30 min, unpaired *t*-test. (**c**) A fluorescence image of an anvil-shaped VD growth cone. White dotted line represents the putative lateral nerve cord. Scale bar, 3 μm. (**d**) Partial illumination of the anvil-shaped growth cone. The merged image (upper: Red; 0 min, Blue; 30 min, lower: Red; 30 min, Blue; 60 min) was attached. Scale bar, 3 μm. (**e**) Temporal centroid plots of the illuminated anvil-shaped growth cones. Second illuminations were performed either anteriorly or posteriorly. Each experimental trial is shown with the same colored line. Error bars, 98% CI of the mean (*n* = 10).

Growth cones of VD neurons in anesthetized L2 transgenic mutant worms were visualized with fluorescence of mCherry. Movement of the growth cones was analyzed by determining the centroids of growth cones (Fig. S7a–c). Before illumination, ruffling VD growth cones showed slow movement in different directions (0.90 ± 0.26 μm h^−1^, *n* = 10). A part of the growth cones was illuminated with blue laser light under the confocal microscope in a pulse-like manner (2 s min^−1^). Time lapse imaging with light stimulation showed growth cone attraction toward the illuminated side (Fig. 5a and Movie S2). To ascertain the extent of light-induced attraction, centroids of each illuminated growth cone were plotted over time (Fig. 5b and Fig. S7d). The centroids with a positive value represent growth cone attraction toward the illuminated side. The temporal profile of centroid plots revealed clear light-induced attraction in growth cones expressing PA-UNC-40::Venus, especially 15 min after the onset of the illumination, judging from the 98% confidence interval (0.02–0.14 μm). However, blue light illumination to the growth cones without PA-UNC-40::Venus expression did not affect the growth cone movements. Moreover, when *ced-10*, a downstream molecule of UNC-40 signaling^38^,^39^, was deleted, light-induced attraction by PA-UNC-40::Venus was abolished (Fig. S8a,b). After repeated illuminations, a PA-UNC-40::Venus localization pattern changed from a uniform distribution to preferential localization to the illuminated side of the all examined growth cones (Fig. S6a,b). The localization pattern of PA-UNC-40::Venus after repeated illuminations were similar to the ventral localization of UNC-40 corresponding to the endogenous UNC-6 gradient generated by ventral cells^37^. These results suggest that light-induced activation of PA-UNC-40 mimicked the intrinsic property of UNC-40 activation by UNC-6. Collectively, blue laser light illumination enables attraction of the growth cone that expresses PA-UNC-40 in living nematode worms.

A heterogeneous extracellular milieu exists around the growth cone in *C. elegans*. During the extension of VD neurons, VD growth cones stall at two physical barriers, the lateral nerve cord, and the dorsal body wall muscle^28^. Stalled growth cones spread along the barrier and become anvil-shaped. To examine the difference in the motility towards different directions, the anvil-shaped growth cone, which is presumably trapped at the lateral nerve cord (Fig. 5c), was sequentially illuminated on two sides during the observation (Fig. 5d and Movie S3). First, the dorsal side of the anvil-shaped VD growth cone was illuminated. The blue light did not induce any visible attractive response. In contrast, the subsequent anterior illumination triggered marked growth cone intrusion into the illuminated area. These behaviors were visualized by centroid tracking from multiple experiments, showing no readily observable dorsal movement (0–30 min), but showing a gradual shift to the illuminated side along the anterior-posterior axis (30–60 min) (Fig. 5e and Fig. S9). Judging from the 98% confidence intervals, the examined growth cones were attracted to the illuminated side especially 13 min after the onset of the illumination (0.01–0.59 μm). It is noteworthy that centroid plots with a positive value represent attraction to the illuminated side. As described above, illumination to the anvil-shaped growth cone evoked either attractive or non-attractive response, depending on the illumination position. These results demonstrate that the PA-UNC-40 system enables analysis of the growth cone behavior within intrinsic extracellular environments by photo-manipulation *in vivo*.

## Discussion

Light-induced oligomerization system can activate the axon guidance receptor DCC in a form of CRY2 fused molecule, PA-DCC, as demonstrated herein. Endogenous DCCs normally form oligomer upon binding with the attractive guidance molecule, netrin-1. Recent X-ray crystal structural analysis showed that netrin-1 serves as a scaffold for local DCC assembly via its two receptor-binding sites^25^. However, it remains unclear whether netrin-1 binding induces a conformational change in DCC for the activation. In this study, we demonstrated for the first time that DCC oligomerization is sufficient for evoking the growth cone turning. The results suggest that the oligomerization is a key step in the DCC activation, which is consistent with the fact that overexpression of DCC typically engenders their activation without netrin-1^26^. Additionally, *C. elegans* UNC-40 was activated with the same light-induced receptor oligomerization system, which constitutes direct evidence showing that a common activation mechanism exists in DCC homologs among species. To our surprise, light-induced local activation of DCC eventually attracted growth cones without mechanically tethered guidance cue molecules. Indeed, the physical attachment of netrin-1 to the substrate, extracellular matrix or dishes, plays a key role in the induction of the attractive response^40^. However, when we considered that PA-DCC molecules are not physically tethered to any substrate during the light-induced attraction, our experimental finding suggests that the substrate attachment of netrin-1 is not necessary for the growth cone attraction. Nevertheless, it might be important at a physiological level of netrin-1 concentration.

One benefit of our strategy is its applicability to an *in vivo* system, enabling the analysis of growth cone behavior in living animals. We demonstrated that light illumination can attract the VD growth cone expressing PA-UNC-40 in living worms. It is particularly interesting that the dorsal side illumination to the anvil-shaped VD growth cone did not induce attractive response. The shape and position of the illuminated growth cone suggested that the light-induced attractive signaling with PA-UNC-40 was insufficient to cross the lateral nerve cord where the dorsal movement of the growth cone was physically hampered. Given that the extent of light-induced downstream signaling was not different in illuminations on either side, the lateral cord was able to impede the VD growth cone advancement physically, thereby restricting its motility. The developed photo-manipulation system can analyze the growth cone behavior in the presence of surrounding physical barriers in living animals. The possibility exists that it will further enable us to examine, in a site-specific manner, how extracellular environments permit or inhibit growth cone entry to achieve highly precise neural wiring in a specific stage of the development.

Optical modulation of biological phenomena in living cells is sometimes accompanied by adverse effects of the light itself: photo-toxicity and photo-thermal effects. In earlier observations using a confocal laser microscopic system, for instance, slowed migration of growth cones in *C. elegans* was detected with excessive light exposure. We also observed axon retraction in our developed system with intentionally longer illumination time than the standard protocol described, especially in the *C. elegans* growth cone (data not shown), which suggests the susceptibility of the mutant *C. elegans* growth cones to light-induced damage. Nevertheless, the photo-toxic effect is avoidable by optimizing the illumination protocol because we rarely observed axon retraction or slowed migration during the assays in the established illumination condition. Regarding the photo-thermal effect, it was recently reported that light-induced warming with near-IR laser can repel growth cones^41^. For effective local heating with laser light, high light dose (100 mW μm^−2^ for more than 10 min) and long wavelength (>700 nm) are necessary. In contrast, such effects are negligible in our method because the developed photo-reactive molecules are activated with low dosage of light (0.07 nW μm^−2^ for 5 s every 5 min) and shorter (425–440 nm) wavelength. Moreover, no repulsive response was detected with light illumination alone. To maximize the light-induced attractive response, it is important to seek the best light condition which enables both effective photo-activation and reduced light-adverse effects. Such a condition can be achieved by changing the exposure time, intensity interval, and the illumination position. Because CRY2 is activatable with two-photon excitation using the near-IR light (830–980 nm)^12^, it is possible to manipulate the growth cone behaviors optically deep inside the living tissue, with careful consideration of the adverse effects described above.

DCC is only one of the various guidance receptors involved in the neural circuit formation. During nervous system development, numbers of ligand/receptor signaling pathways dynamically cooperate mutually to achieve highly complicated neural wirings without errors^3^. Considering that the light illumination activates the receptor of interest in our system specifically, it is also possible to expand the system for photo-controlling the function of other guidance receptors with similar activation mechanisms, such as UNC-5, which mediates short-range repulsion in a homodimer^42^. It is possible to activate different guidance receptors separately with different wavelength light in a spatiotemporal manner if a red-shifted light oligomerizing module is developed. The multiple photo-activatable guidance receptor system will allow for disentangling the roles of each receptor signaling pathway in growth cone behavior, thereby giving us new insights into the dynamics of combinatorial signaling effects on axon guidance in living animals.

In summary, DCC connected with CRY2 (PA-DCC) is activatable with blue-light illumination by the photo-clustering of fused CRY2. Results show that the local activation within growth cones changed the direction of the growth in both chick DRG neuron and *C. elegans* VD neuron. This is the first method that enables optical manipulation of growth cone behavior *in vivo*. The system is readily applicable to other guidance receptors, which are activated by their oligomerization, thereby expanding the range of experimental tools to analyze the guidance mechanisms, particularly in the developing nervous system. Further optimization of the photo-manipulation system will enable the optical modulation of axon pathfinding in living animals, which can contribute considerably to the development of regenerative therapies for neurodegenerative disorders.

## Materials and Methods

### Strains and culture

The *C. elegans* nematode was maintained using standard techniques^43^. The following alleles and transgenes were used for the assays; EG1285 *lin-15(n765); oxIs12[Punc-47::gfp; lin-15(+)]* X, JN1900 *unc-5(e53)* IV; *unc-6(ev400)* X; *peEx1900[Punc-47::PA-UNC-40::venus; Punc-47::mCherry; Plin-44::gfp]*, and JN1901 *unc-5(e53)* IV; *unc-6(ev400)* X; *peEx1901[Punc-47::mCherry; Plin-44::gfp]*, JN1902 *ced-10(n1993) unc-5(e53)* IV; *unc-6(ev400)* X; *peEx1900[Punc-47::PA-UNC40::Venus; Punc-47::mCherry; Plin-44::gfp].* Extrachromosomal arrays were generated by microinjection as described^44^ using *Plin-44::gfp* as a coinjection marker. The double mutant was confirmed by uncoordinated phenotypes and PCR genotyping.

### Plasmid construction

The cDNAs of DCC and FAK were amplified from *Mus musculus* brain cDNA mixture (GenoStaff Co. Ltd., Tokyo, Japan). The UNC-40 cDNA was obtained via RT-PCR from total *C. elegans* RNA. The CRY2 cDNA was amplified from pGal4BD-CRY2 (Addgene, MA, USA). Additional point mutation for CRY2 (D387A) was induced by mutagenic complement oligo single-stranded DNA pairs. The PCR products (DCC, CRY2, CRY2 (D387A), FLAG tagged FAK) were subcloned into pcDNA4 vector (Invitrogen Corp., CA, USA) or pcDNA3.1 vector (Invitrogen Corp.). For expression in primary cultured chick DRG neuron, cDNAs were subcloned into the pCAGGS vector (provided by J. Miyazaki, Osaka University, Osaka, Japan). In experiments for *C. elegans*, cDNAs were cloned under the *unc-47* promoter by the GATEWAY system (Invitrogen Corp.) as described^45^. All primers used for this study are listed in Table S1.

### Cell culture and transfection

Human embryonic kidney (HEK293) T cells were cultured in Dulbecco’s Modified Eagle Medium (DMEM, high glucose; Wako Pure Chemical Inds. Ltd., Japan) supplemented with 10% Fetal Bovine Serum (FBS; Gibco, CA, USA), 100 unit mL^−1^ penicillin and 100 μg mL^−1^ streptomycin (Gibco) at 37 °C in 5% CO_2_. Cells were transfected transiently with expression vectors using Lipofectamine 2000 (Invitrogen Corp.) according to the manufacturer’s protocol.

For growth cone turning assays with DRG neurons from E9 chick embryos, the expression vectors were introduced to the dissociated neurons by electroporation (neon; Invitrogen Corp.). The neurons were diluted in RPMI1640 medium (Gibco) supplemented with 10% FBS and 40 ng mL^−1^ NGF (Promega Corp., WI, USA). To generate neuron balls, 20,000 neurons/50 μL drops were plated on the lids of 100 mm dishes filled with 7 mL of water. Three days after hanging drop cultures at 37 °C in 5% CO_2_, neuron balls in each drop were transferred individually to glass bottom dishes coated with poly-D-lysine and laminin (Invitrogen Corp.) in RPMI1640 medium supplemented with 10% FBS and 20 ng mL^−1^ NGF.

### Immunocytochemistry

HEK293T cells were fixed with 4% paraformaldehyde in phosphate buffer saline (PBS) at 37 °C for 20 min and washed with PBS. The cells were permeabilized with 0.2% Triton X-100 in PBS at room temperature for 20 min. The cells were blocked with 0.2% gelatin from cold water fish skin (FSG; Sigma-Aldrich Corp., MO, USA) in PBS for 1 h at room temperature or overnight at 4 °C. The blocked cells were incubated with the indicated primary antibodies and immunostained with the appropriate secondary antibodies labeled with fluorescent dyes in PBS (0.2% FSG). All antibodies used in the analysis were listed in Supplementary Table S2. The cells were mounted with FluorSave (Calbiochem) on a slide glass. Images were taken on a confocal fluorescent microscope (FV-1000D; Olympus Corp., Tokyo, Japan).

For DRG neurons, equal amount of 8% paraformaldehyde 8% Sucrose in PBS was added to the medium at room temperature for 20 min. The neurons were permeabilized with 0.1% Triton X-100 in PBS at room temperature for 10 min. The neurons were blocked with 5% goat serum (Vector Laboratories, CA, USA) 0.01% Triton X-100 in PBS for 1 h at room temperature. The blocked neurons were incubated with the indicated primary antibodies in Can Get Signal Immunoreaction enhancer solution (TOYOBO, Osaka, Japan) and immunostained with the appropriate secondary antibodies labeled with fluorescent dyes in PBS (1% goat serum, 0.01% Triton X-100). All antibodies used in the analysis were listed in Supplementary Table S2. The neurons were mounted with ProLong Diamond (Invitrogen Corp.). Images were taken on a confocal fluorescent microscope (FV-1000D).

### Western blot analysis

Transiently transfected HEK293T cells were stimulated with LED light (0.05–5 mW cm^−2^ at 440 nm, Optocode Corp., Tokyo, Japan) for 1–15 min before cell collection. The other cells were incubated with or without recombinant mouse netrin-1 (200 ng mL^−1^; R&D Systems, MN, USA) for 15 min in the dark.

For phosphorylation detection assays, cells were collected with ice-cold PBS and lysed in the PLC buffer (50 mM HEPES, pH 7.5, 150 mM NaCl, 10% glycerol, 1% Triton X-100, 1.5 mM MgCl_2_, 1 mM EGTA, 10 mM NaPPi, 100 mM NaF, 1 mM Na_3_VO_4_ and protease inhibitor Complete (Roche, Switzerland)). The lysates were boiled with the sample buffer (10% SDS, 25% glycerol, 5% 2-mercaptoethanol, 0.02% Bromophenol Blue, 250 mM Tris–HCl, pH 7.6) at 95 °C for 5 min.

For analysis of oligomerization, the co-IP protocol was designed to probe transient or dynamic protein complex based on the method for detecting CRY2-CIB1 complex^22^. One microliter of mouse anti-V5 antibody was pre-incubated in a 50 μL of Protein G sepharose (GE Healthcare) diluted by PLC buffer at 4 °C for 2 h. Cells were collected with the ice-cold PLC buffer and subjected to centrifugation at 4 °C for 2 min. The supernatant was incubated with the pre-incubated anti-V5 antibody at 4 °C for 30 min with rotation. The collected sample was denatured with the sample buffer at 95 °C for 5 min. Protein G sepharose were removed by centrifugation.

The samples were separated by SDS polyacrylamide gel electrophoresis and transferred onto nitrocellulose membrane (GE Healthcare). The membrane was blocked with 1% skimmed milk or 2% ECL Advance Blocking Agent (GE Healthcare) in Tris-buffered saline containing Tween-20 (TBS-T; 150 mM NaCl, 0.05% Tween-20, 50 mM Tris-HCl, pH 8.0). The membrane was reacted with the indicated primary antibodies and with the appropriate secondary antibodies labeled with horseradish peroxidase (Supplementary Table S2). The immunoblot bands were detected using SuperSignal West Femto Substrate (Thermo Scientific, IL, USA) with an image analyzer (LAS-1000 plus; Fuji Photo Film Co. Ltd., Tokyo, Japan).

### Growth cone turning assays in chick DRG neurons

One day after plating neuron balls, the medium was exchanged with Leibovitz’s L-15 medium (Gibco) supplemented with N-2 (Gibco), 20 ng mL^−1^ NGF, 750 μg mL^−1^ bovine serum albumin (Gibco) and 40 μM Sp-cAMPS (Calbiochem). After incubation for 30 min, neurons were observed by an inverted microscope (IX81; Olympus Corp.) with objective lens (UPlanApo 100×; Olympus Corp). An area within one side of the growth cone was illuminated with a blue light (metal halide lamp with 425–440 nm filter, 8 nW sample-site beam power at 440 nm, for 5 s every 5 min pulses) while time-lapse differential interference contrast (DIC) images of the growth cone were acquired with a CCD camera (CoolSNAP ES; Roper Scientific Inc., NJ, USA). The size of the illuminated region (approx. 5 μm diameter) was modulated by a pinhole placed in the light path. The illumination methods were based on the previous reports^46^,^47^. Growth cone turning was assessed by tracing the growth cone center defined as the intersection of the extrapolated line from the axon and the distal edge of the growth cone central domain. The original direction of axon extension was defined by a straight line connecting the positions of the growth cone at the onset and 5 min before photo-activation. The direction after the stimulation was defined as a straight line connecting the positions of the growth cone at the onset and at the end of repetitive stimulation. The growth cone turning angle was defined by the angle between the original direction and the direction after the stimulation. Only growth cones with net extension over 6 μm were included for analysis.

### Analysis of axon guidance defects in *C. elegans*

VD/DD neurons in *C. elegans* were visualized with an *Punc-47::mCherry* or *Punc-47::GFP* transgene, expressed in all GABAergic neurons^34^. VD/DD axons were scored as guidance defective when they failed to reach the dorsal nerve cord, or branched or turned at an angle greater than 45°.

### Analysis of growth cone attraction in *C. elegans*

VD neurons were visualized with an *Punc-47::mCherry* transgene. Adult worms were lysed to collect eggs with an alkaline-bleach solution and suspended in the M9 buffer and incubated until L1 larva hatch. L1 arrested worms were placed onto NGM plates seeded with OP50 and grown at 20 °C. L2 worms were transferred into M9 droplet on the glass bottom dish and immobilized with 0.02% levamisole (Sigma-Aldrich Corp.). A 5% agarose pad was placed on the droplet. Growth cones were observed with a confocal microscope within 3 h after the immobilization. Ruffling of lamellipodia in growth cones were examined 10 min before illumination to rule out stalled growth cones. The illumination power used for stimulation of PA-DCC was 0.1% with 20 mW LD 488 nm laser light at a speed of 2.0 μs pixel^−1^. Centroids of growth cones and intensity of Venus fluorescence were calculated using ImageJ software.

## Additional Information

**How to cite this article**: Endo, M. *et al*. Optogenetic activation of axon guidance receptors controls direction of neurite outgrowth. *Sci. Rep.* **6**, 23976; doi: 10.1038/srep23976 (2016).

## Supplementary Material

Supplementary Information

Supplementary Movie S1

Supplementary Movie S2

Supplementary Movie S3
