# Supplementary material for: Optogenetic activation of axon guidance receptors controls direction of neurite outgrowth
Source: Sci Rep. 2016 Apr 7;6:23976. doi: 10.1038/srep23976 (PMC4823752; doi:10.1038/srep23976)
Supplement: Supplementary Information [file srep23976-s1.pdf]

Supplementary Materials for

Optogenetic activation of axon guidance receptors controls direction of neurite outgrowth

M. Endo, M. Hattori, H. Toriyabe, H. Ohno, H. Kamiguchi, Y. Iino, T. Ozawa

The file includes:

Figure S1. Localization of DCC-containing molecules in HEK293T cells.
Figure S2. Reversibility of PA-DCC activation.
Figure S3. Localization of PA-DCC and PA-DCC (D387A) in chick DRG neuron.
Figure S4. Time course turning angle analysis of growth cones with repositioned illuminations.
Figure S5. Axon guidance defects of worms used for this study.
Figure S6. Localization of PA-UNC-40 in *C. elegans* VD neuron.
Figure S7. Procedures for growth cone centroid analysis in *C. elegans* VD neurons.
Figure S8. Illumination on VD growth cones in *ced-10(n1993); unc-5(e53); unc-6(ev400)* with PA-UNC-40::Venus expression.
Figure S9. Temporal centroid plots of the illuminated anvil-shaped growth cone.
Table S1. All primers used for this study.
Table S2. Antibody list used in this study.
Movie S1. *In vitro* axon guidance with PA-DCC.
Movie S2. *In vivo* growth cone attraction with PA-UNC-40.
Movie S3. Blue light exposure to the anvil-shaped growth cone expressing PA-UNC-40.

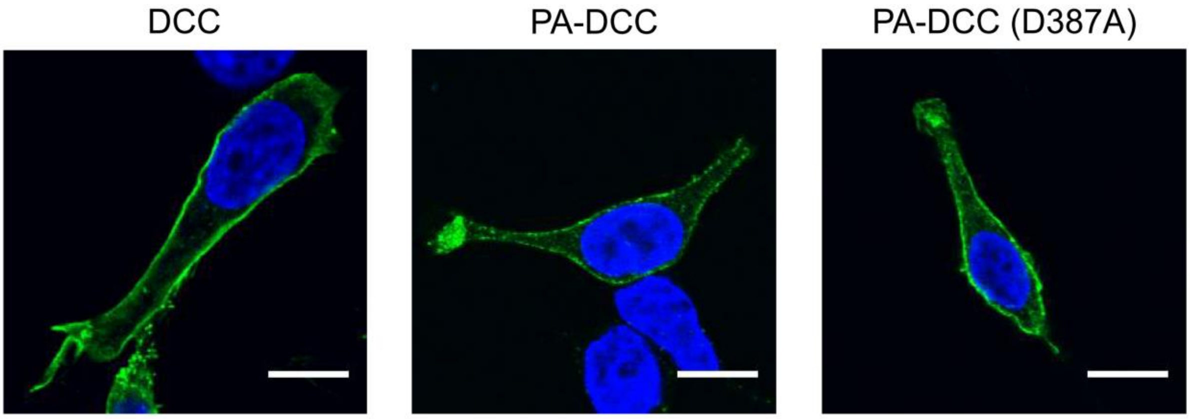

Figure S1.

Localization of DCC-containing molecules in HEK293T cells. Each molecule was immunolabeled with Alexa488 (green) using V5-epitope tag in fixed cells. Nucleus was stained with Hoechst33342 (blue). Scale bar, 10 μm.

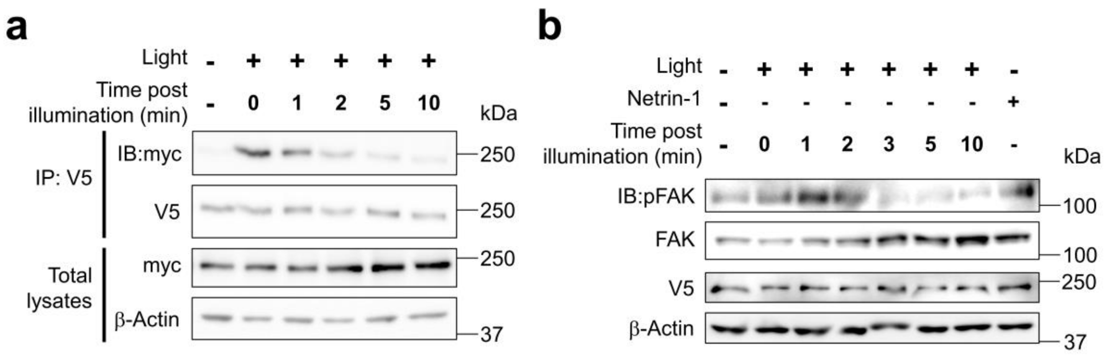

Figure S2.

Reversibility of PA-DCC activation. (a) Dissociation profiles of PA-DCC oligomer. Light-illuminated cells were incubated for each indicated time. The proteins were isolated by IP with anti-V5 antibody. Co-immunoprecipitated myc-tagged PA-DCC was visualized using Western blotting. The figure is a representative data of Fig. 2k. (b) Dephosphorylation of FAK after light illumination. Cells were illuminated and incubated in a dark condition for the indicated time. The cell components were analyzed with each antibody. The figure is a representative data of Fig. 2l.

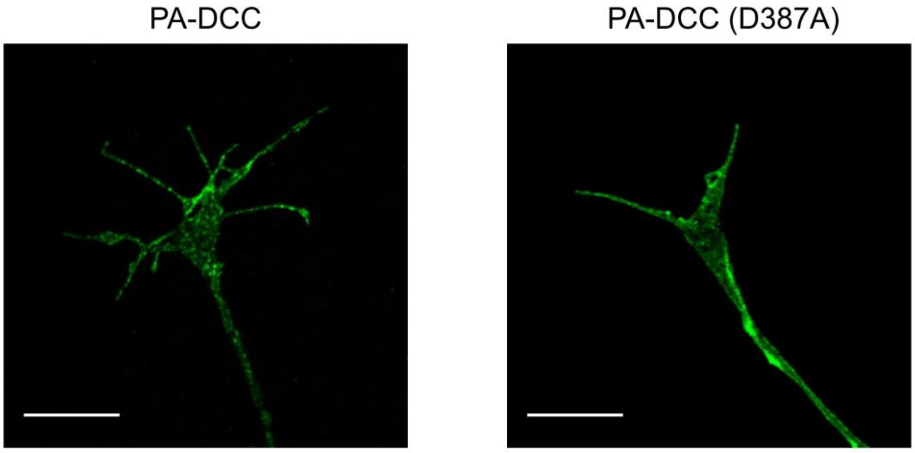

Figure S3.

Localization of PA-DCC and PA-DCC (D387A) in chick DRG neuron. Each molecule was immunolabeled with Alexa488 (green) using V5-epitope tag in fixed neurons. Scale bar, 10 μm.

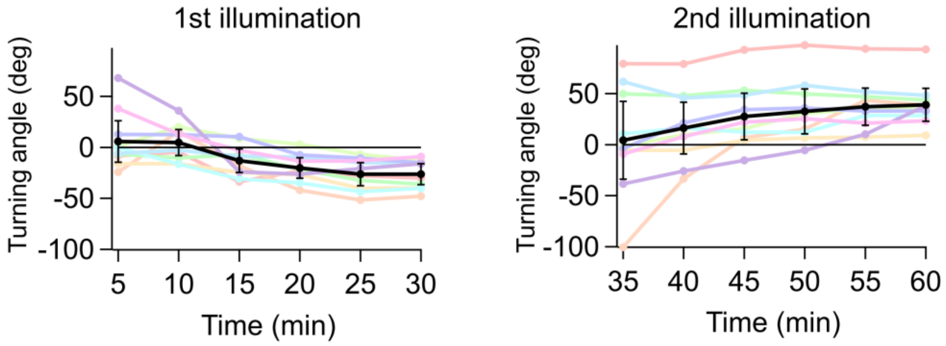

Figure S4.

Time course turning angle analysis of growth cones with repositioned illuminations. Each data is represented by a colored line. Averaged data are shown as black. Error bars, 98% CI of the mean (n = 10).

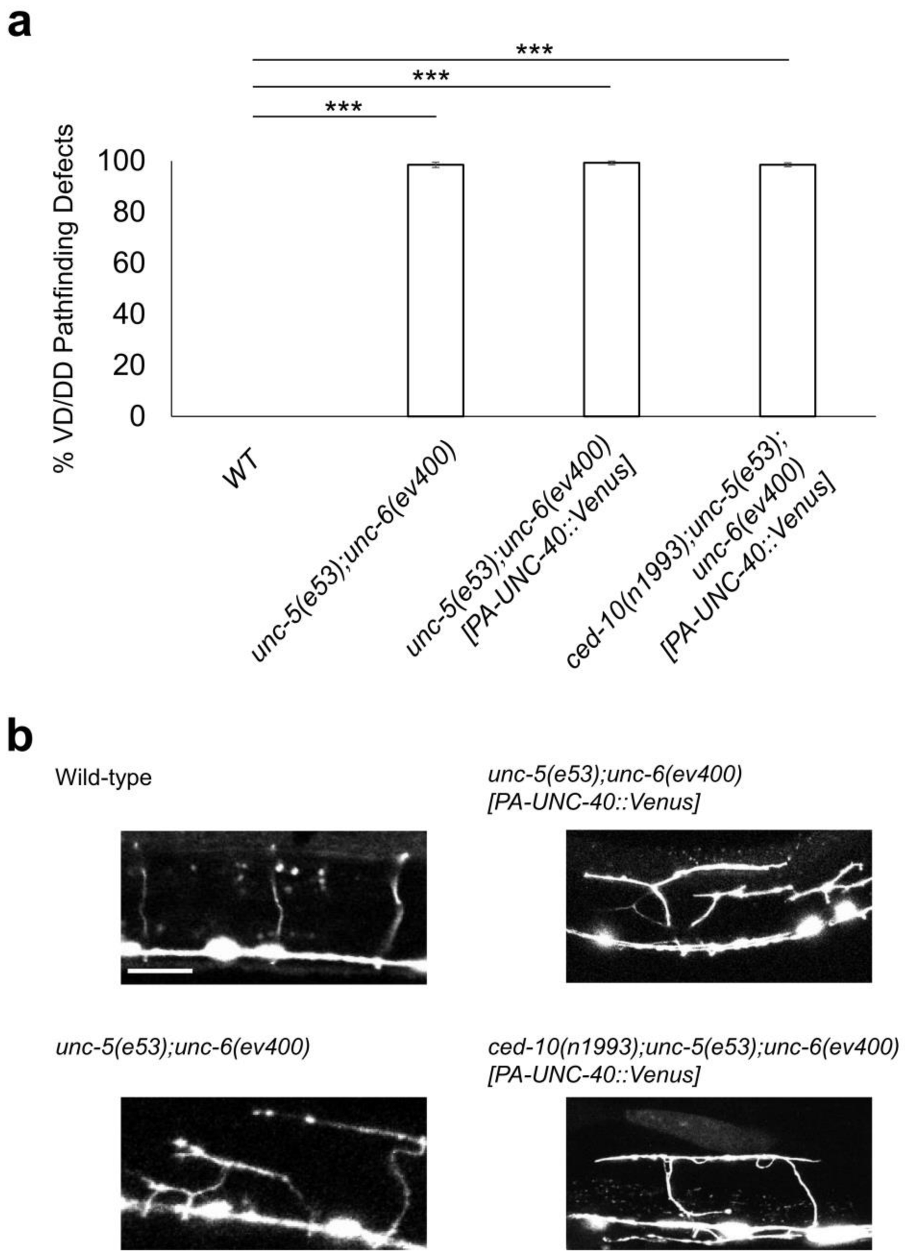

Figure S5.

Axon guidance defects of worms used for this study. (a) Percentage of VD/DD axon pathfinding defects. Error bars, SEM (*n* > 100). Statistical significance was determined by one-way ANOVA followed by Dunnett's test for multiple comparison, ****P* < 0.005. (b) Fluorescence micrographs of L4 worms showing VD/DD axons. Dorsal side is up, anterior is to the left. Scale bar, 20 μm.

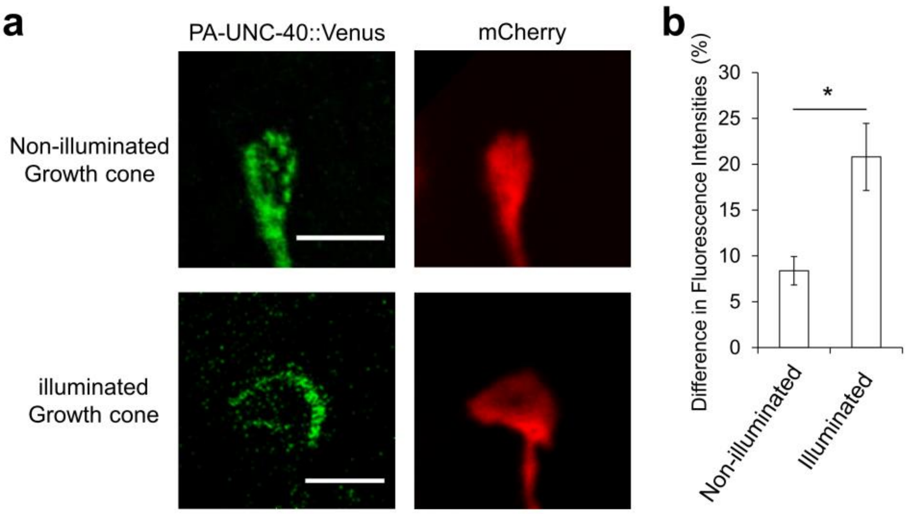

Figure S6.

Localization of PA-UNC-40 in *C. elegans* VD neuron. (a) Fluorescence images of *unc-5(e53); unc-6(ev400)* VD growth cones expressing PA-UNC-40::Venus and mCherry. Lower images were taken after the illumination to the right side of the growth cone (2 s min^{-1} pulses for 30 min). Scale bar, 3 μm. (b) Analysis of the PA-UNC-40::Venus distribution on the growth cone. The difference in fluorescence intensities was defined as the difference of the total Venus fluorescence intensities in each side relative to the total Venus intensity in the both side. Then, the obtained result was normalized by that of mCherry fluorescence. Mean distribution inequities were shown as scores. Error bars, s.e.m (n = 5). *$P < 0.05$, unpaired *t*-test.

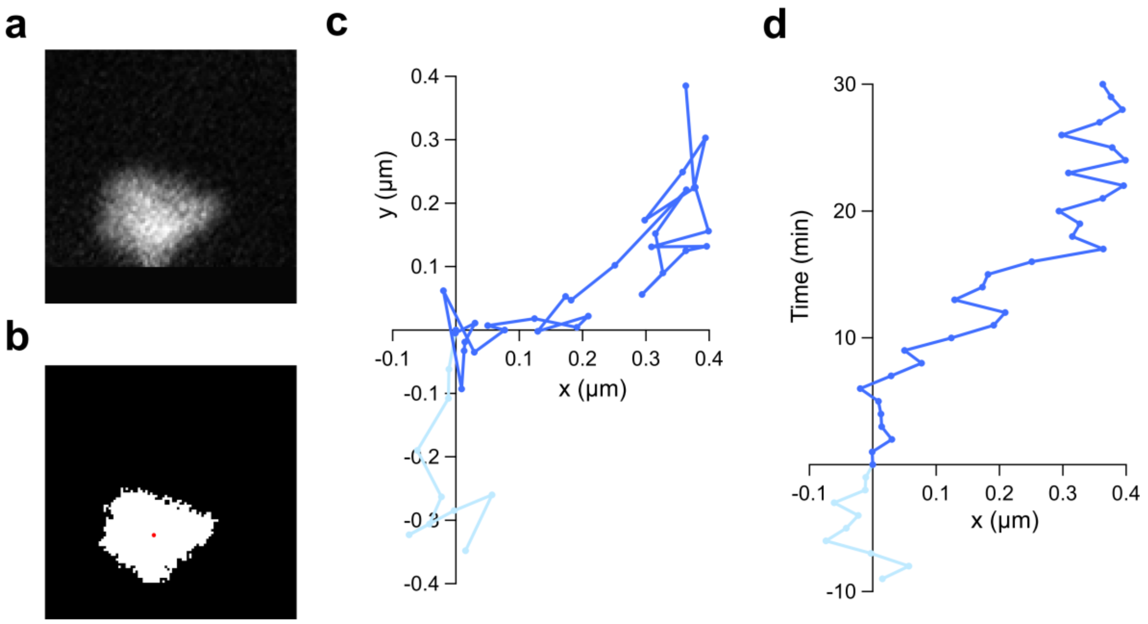

Figure S7.

Procedures for growth cone centroid analysis in *C. elegans* VD neurons. (a) Representative image of the trimmed growth cone in Fig. 5a. The trimmed growth cone was rotated and inverted to put the illuminated side at the right and the axon at the bottom. (b) Binarized image of Fig. S7a. The red dot indicates the position of the centroid. (c) Centroid plots of the growth cone in Fig. S7a. The position of centroid at 0 min was defined as the origin. (d) Centroids plots against time modified from Fig. S7c. The centroid displacement from the origin along x-axis (Fig. S7c) was displayed against time-axis.

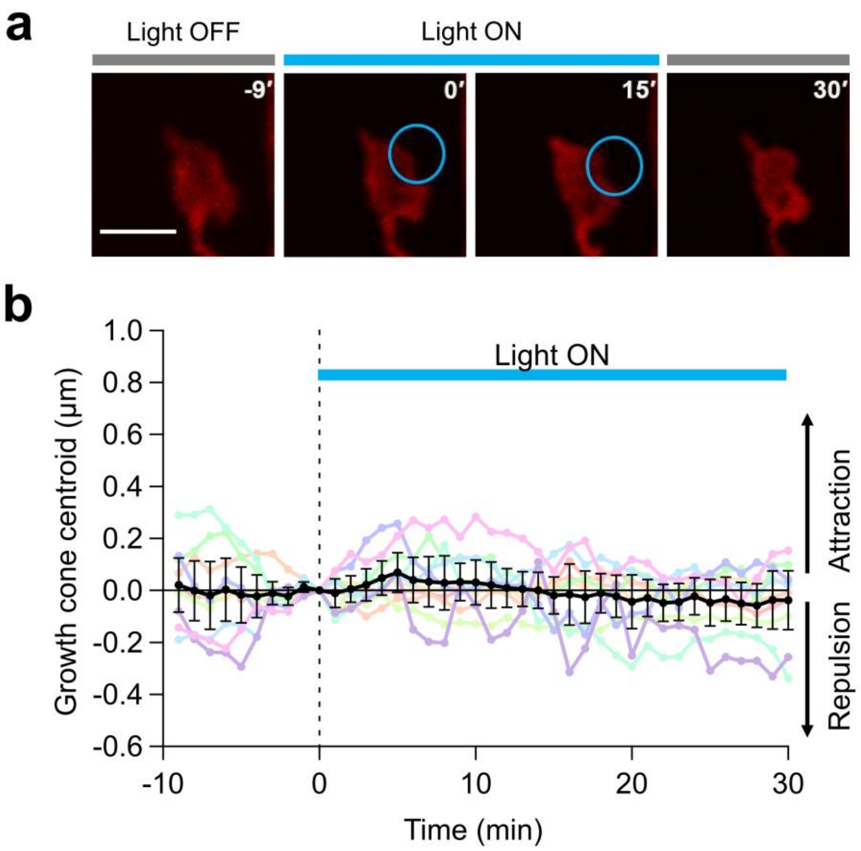

Figure S8.

Illumination on VD growth cones in *ced-10(n1993); unc-5(e53); unc-6(ev400)* with PA-UNC-40::Venus expression. (a) Time lapse images showing the growth cones with periodically illuminated with blue light. Illuminated regions with blue laser light (2 s min^{-1} pulses, 488 nm) were represented as blue circles. Scale bar, 3 μm. (b) Temporal centroid plots of periodically illuminated growth cones. Each experimental trial is shown with the same colored line. Averaged data are shown as black. The blue bar indicates the illumination period. Error bars, 98% CI of the mean. (*n* = 10).

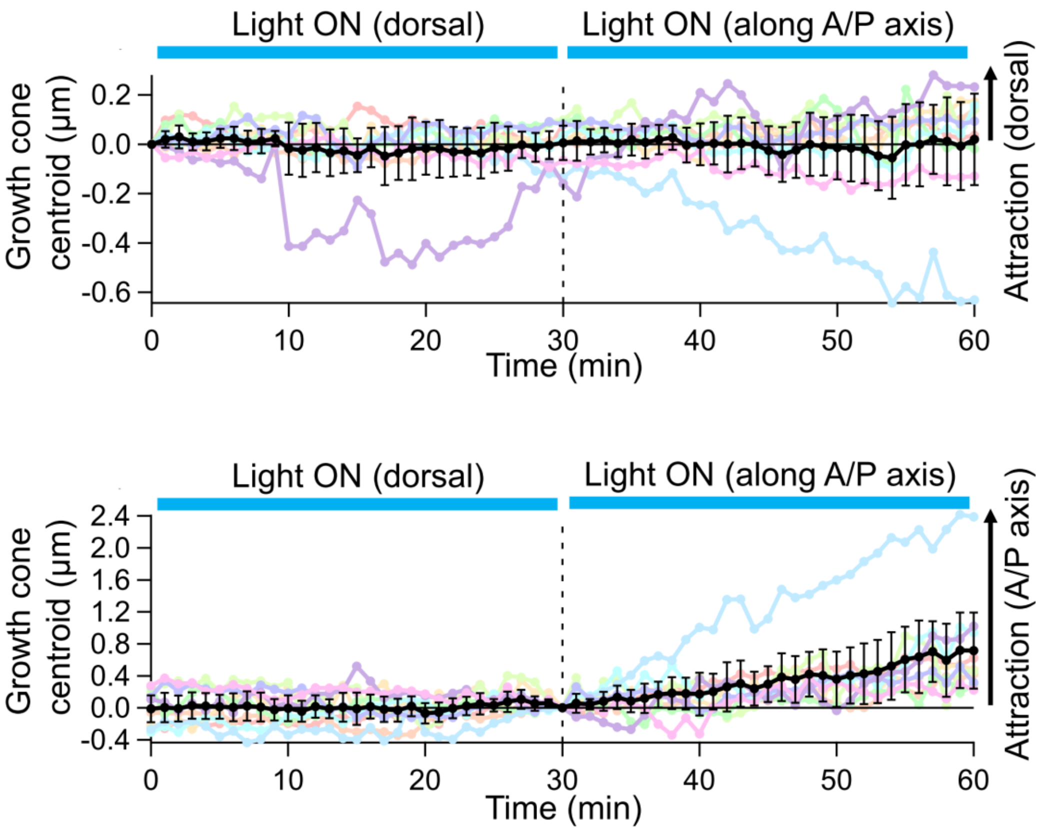

Figure S9.

Temporal centroid plots of the illuminated anvil-shaped growth cone. Analyzed growth cones were the same in Fig. 5e. Each experimental trial is shown with the same colored line. Averaged data are shown as black. Blue bars indicate illumination periods. Error bars, 98% CI of the mean (n = 10).

Primer	Primer sequence
Dcc-U1	CGGCCCCGAAGGTGTTGG
Dcc-L1	CAGAAGATCTCAACGTTTTGGC
EcoRV-Dcc-U	GCAGATATCATGGAGAATAGTCTTGGATGTG
Dcc-NotI-L	CGAGCGGCCGCCAAAGGCAGACCCTGTGATGG
Cry2-V5-L	AGGGTTAGGGATAGGCTTACCTTTGCAACCATTTTTTCCCAAAC
Cry2-myc-L	TCTTCTGAGATGAGTTTTTGTTCTTTGCAACCATTTTTTCCCAAAC
V5-XbaI-L	CCCTCTAGATTTCTCGAGCTTCACGTAGAATCGAGACCGAGGAGAGGGTTAGGGATAGG CTTAC
myc-XbaI-L	CCCTCTAGATTTCTCGAGCTTCACAGATCCTCTTCTGAGATGAGTTTTTGTTC
V5-ClaI-XbaI-L	AAATCTAGATAATTTATCGATTCCCGTAGAATCGAGACCGAGG
ClaI-mCherry-U	AAAATCGATATGGTGAGCAAGGGCGAGG
mCherry-XbaI-U	AAATCTAGATTACTTGTACAGCTCGTCC
NheI-FLAG-U	CTGGCTAGCGATGGACTATAAGGATGACGACG
FLAG-EcoRV-L	CTGGATATCTTTATCGTCGTCATCCTTATAGTCC
Ptk2-U1	ACAGATACCTAGCATCTAGC
Ptk2-L1	CAGAAAGCCACGTTCAATGG
EcoRV-Ptk2-U	AAAGATATCATGGCAGCTGCTTATCTTGACC
Ptk2-NotI-L	CGAGCGGCCGCCTCAGTGTGGCCGTGTCTGC
XhoI-mCherry-U	CCGCTCGAGAAATCTAGAGATGGTGAGCAAGGGCGAGG
mCherry-SacII-L	GAACCGCGGTTACTTGTACAGCTCGTCCATG
UNC-40-U1	AGAAATGATTTTGCGACATTTC
UNC-40-L1	TGCAAAATTAATAATTCAAATTATC
NheI-UNC-40-U	AAAGCTAGCAAAAATGATTTTGCGACATTTCGG
UNC-40-NotI-L	AAAGCGGCCGCCCTTATCCATACTCGTCTCAAA
V5-Venus-U	CCTCGGTCTCGATTCTACGATGAGTAAAGGAGAAGAACTT
V5-Venus-L	CGTAGAATCGAGACCGAGGAGAGGGTTAGGGATAGGC
Venus-EcoRV-L	AAAGATATCTCATTTGTATAGTTCATCCATGCC

Table S1. All primers used for this study.

Antibody	Company	Catalogue No.	Application (Dilution)
V5	novex	R960-25	WB (1:4000), IC (1:1000-4000)
myc	Cell Signaling Technology	2276	WB (1:4000)
βActin	Sigma-Aldrich Corp.	A1978	WB (1:4000)
FLAG	Sigma-Aldrich Corp.	F3165	WB (1:4000)
Phospho-FAK [pY397]	Invitrogen Corp.	44-624G	WB (1:2000)
Phospho-FAK [pY397]	GeneTex	GTX61795	IC (1:500)
PLCγ-1	Upstate Biotechnology	05-163	WB (1:2000)
Phospho-PLCγ-1 [pY783]	Cell Signaling Technology	2821	WB (1:2000)
Mouse IgG labeled with horseradish peroxidase	GE Healthcare	NA931	WB (1:5000)
Rabbit IgG labeled with horseradish peroxidase	GE Healthcare	NA934	WB (1:5000)
Alexa-Fluor 488 conjugated donkey anti-mouse IgG	GE Healthcare	A-21245	IC (1:2000)
Alexa-Fluor 647 conjugated goat anti-rabbit IgG	GE Healthcare	A-21202	IC (1:2000)

Table S2. Antibody list used in this study.

Movie S1.

In vitro axon guidance with PA-DCC. A part of chick DRG growth cone expressing PA-DCC was illuminated with blue light (5 s pulse every 5 min). Blue circles represent the illuminated region. Scale bar, 10 μm.

Movie S2.

In vivo growth cone attraction with PA-UNC-40. A part of VD growth cone expressing PA-UNC-40 was illuminated with blue laser (2 s min^{-1} pulses, 488 nm). Blue circles represent the illuminated region. Scale bar, 3 μm.

Movie S3.

Blue light exposure to the anvil-shaped growth cone expressing PA-UNC-40. A part of anvil-shaped VD growth cone expressing PA-UNC-40 was illuminated with blue laser (2 s min^{-1} pulses, 488 nm). Blue circles represent the illuminated region. Scale bar, 3 μm.
